# Utilizing 3D Printing and Distributed Optic Fiber to Achieve Temperature-Sensitive Concrete

**DOI:** 10.3390/ma18091897

**Published:** 2025-04-22

**Authors:** Qiuju Zhang, Yujia Li, Yuefan Huang, Yangbo Li, Yahui Yang, Yutao Hu

**Affiliations:** College of Hydraulic and Environmental Engineering, China Three Gorges University, Yichang 443002, China; 202208150021056@ctgu.edu.cn (Q.Z.); 202008150021038@ctgu.edu.cn (Y.L.); 202208150021014@ctgu.edu.cn (Y.H.); liyangbo@ctgu.edu.cn (Y.L.); 202208590021031@ctgu.edu.cn (Y.H.)

**Keywords:** concrete 3D printing, distributed optical fiber, temperature-sensitive, smart concrete

## Abstract

The distribution of temperature-induced cracks in mass concrete structures is extensive and random, making it difficult for existing detection methods to accurately identify the specific location and initiation time of cracking. Therefore, there is an urgent need for an intelligent, precise, and efficient monitoring approach capable of acquiring real-time information on the evolution of the internal temperature field in concrete structures during their early-age curing process. A novel temperature-sensitive concrete system was developed by synchronously integrating distributed optical fibers with three-dimensional printed concrete (3DPC) to enable both temperature monitoring and signal transmission. To validate the effectiveness of the proposed method, experimental testing and numerical simulations were conducted on cubic 3D-printed fiber-reinforced concrete to analyze the temporal evolution of their internal temperature fields. The results show that, during the system calibration process, the temperature measured by the distributed temperature sensing (DTS) system was highly consistent with the environmental temperature curve, with fluctuations controlled within ±1 °C. In addition, the numerical simulation results closely aligned with the experimental data, with discrepancies maintained within 5%, demonstrating the feasibility of utilizing 3D printing technology to impart temperature sensitivity to concrete materials. This integrated approach offers a promising pathway for advancing smart concrete technology, providing an effective solution for accurate sensing and control of internal temperatures in concrete structures. It holds substantial potential for practical applications in civil engineering projects.

## 1. Introduction

During the construction of mass concrete structures, such as dams, embankments, and bridge piers, the heat generated by the cement hydration process induces temperature stress, which is one of the primary causes of concrete cracking [1,2,3]. Construction conditions and boundary constraints can cause the internal temperature stress in certain regions of the concrete to exceed the permissible stress limits at specific times, thereby increasing the risk of cracking. However, it is important to note that elevated stress levels do not necessarily lead to crack formation. Instances where stress surpasses the allowable threshold do not consistently result in cracking. As a result, the likelihood and spatial distribution of potential cracking are heightened [4]. Although there is a risk of cracking throughout the structure, once a crack forms in one region, stress redistribution may relieve stress in other regions, potentially preventing further cracking. This complex phenomenon, which remains difficult to simulate accurately, contributes to the randomness of crack formation. The random and widespread nature of such cracks poses significant challenges for crack monitoring.

To comprehensively capture cracks induced by temperature variations, traditional concrete crack detection methods include visual inspection, crack width measurement, thermometers, ultrasonic testing, infrared thermography, electromagnetic methods, strain gauges, and sensor-based detection systems. Visual inspection is the most commonly used method, typically involving manual examination of crack morphology and distribution. While it is simple and cost-effective, it is limited to identifying surface cracks and cannot detect cracks that develop deeper within the concrete [5]. Ultrasonic testing utilizes the propagation characteristics of sound waves in concrete to locate cracks and determine their depth, making it suitable for detecting deep cracks. However, its accuracy can be influenced by factors such as concrete density and moisture content [6]. Embedding thermometers in concrete also presents limitations, as their fixed positions prevent comprehensive coverage of potential crack locations. Moreover, data collection often relies on manual operations, which not only lack precision in recording crack locations but are also susceptible to various environmental and human factors [7,8,9]. Infrared thermography indirectly identifies crack locations by detecting surface temperature anomalies. It offers advantages such as nondestructive testing and rapid data acquisition; however, its effectiveness is when detecting deep cracks [10]. Electromagnetic techniques are capable of detecting internal cracks and defects, but their performance can be compromised in areas with dense steel reinforcement [11]. Strain gauges monitor changes in surface strain in real time, which can reflect initiation and development of cracks. Although they are suitable for long-term monitoring, they provide limited information regarding the exact locations and morphology of cracks [12]. In recent years, sensor-based crack monitoring systems have been developed [13], capable of real-time monitoring of crack changes, especially for large-scale and long-term monitoring. However, their high costs and complex technical requirements have hindered widespread adoption [14]. As the limitations of traditional detection methods become increasingly apparent, particularly in detecting deep cracks, monitoring in complex environments, and identifying cracks with random distribution patterns, new technological demands have emerged. Conventional methods often fail to deliver comprehensive, real-time monitoring of internal cracks in concrete, especially when cracks are randomly distributed or located deep within the structure. These methods cannot accurately capture the dynamic changes in crack development. Therefore, in response to this issue, smart concrete technology offers an effective solution [15]. By integrating sensing and monitoring capabilities directly into the concrete material, smart concrete offers a more efficient and accurate system for detecting and tracking randomly distributed cracks in real time.

Smart concrete is a multifunctional material that incorporates smart components into the conventional concrete matrix, endowing the material with self-sensing, self-regulating, and self-healing capabilities [16]. Examples such as self-sensing, self-regulating, and self-healing concrete have laid the foundation for further research. Self-sensing concrete [17,18] exhibits various self-sensing properties such as pressure sensitivity, temperature sensitivity, and magnetic sensitivity. In self-sensing concrete, smart materials such as optical fibers [19,20] or carbon fibers [21,22] are typically embedded. These embedded materials enable the detection of temperature variations and structural changes, facilitating real-time monitoring of the concrete’s condition. For instance, self-sensing concrete can detect temperature fluctuations and identify stress or crack formation induced by thermal effects. This critical functionality provides essential data for the structural health monitoring and safety assessment of engineering structures [23].

Currently, the application of optical fiber technology in monitoring concrete cracks, shrinkage [24,25], and other factors has been extensively investigated. By embedding optical fibers within concrete, researchers can monitor real-time strain, temperature variations, and the initiation and propagation of cracks. This has led to significant advancements in areas such as design and construction [26], inspection and maintenance [27], and earthquake disaster mitigation [28], fostering the development of temperature-sensitive concrete (TSC). However, to fully exploit the data collection capabilities of distributed optical fibers, high-precision and highly sensitive acquisition equipment is required. Traditional construction techniques, which are complex and susceptible to interference during installation, often struggle to maximize the advantages offered by high-sensitivity temperature monitoring systems. As an emerging smart construction method, 3DPC offers an effective solution to this challenge. Through digitally controlled, layer-by-layer printing, 3DPC enables the transformation of digital models into physical components with high precision [29]. The rational application of 3D printing in construction enhances mechanization and efficiency, reduces dependence on manual labor [30], and offers additional advantages, including the ability to fabricate complex geometries [31] and customized components. This approach also improves precision and accuracy [32] and reduces material waste [33,34,35]. By integrating distributed optical fibers and 3DPC technology, the resulting temperature-sensitive concrete system (TSCS) allows for temperature monitoring anywhere and within the concrete structure.

Due to the opaque nature and complex internal environment of concrete, conventional temperature monitoring methods struggle to provide continuous, full-field, and spatially resolved measurements of internal temperature evolution. There is an urgent need for an effective approach capable of real-time, distributed sensing of temperature field development during the 3D printing process. To address this challenge, an innovative method for fabricating TSC is proposed, which integrates distributed optical fibers with 3DPC technology to enable real-time monitoring of the internal temperature history of concrete structures. By synchronously embedding optical fiber sensor units during the printing process, this method accurately captures dynamic changes in temperature distribution, providing reliable data support for temperature field analysis. To validate the effectiveness of the proposed approach, experimental tests and numerical simulations were conducted on 3D-printed fiber-reinforced concrete cubes. In the experiments, the temperature variations of the concrete specimens during the curing process were measured, revealing the temporal evolution of the internal temperature field. Simultaneously, numerical simulations based on heat conduction models were performed to provide theoretical insights into temperature field variations. The simulation results were compared with experimental data to assess the accuracy and feasibility of the proposed method, offering both theoretical and practical foundations for the application of temperature-sensitive concrete technology.

## 2. Design Principle of Temperature-Sensitive Concrete System

### 2.1. Principles of the Distributed Temperature System

**Figure 1:**
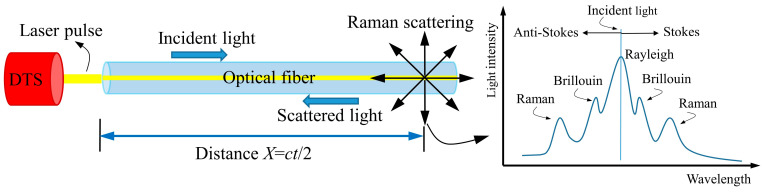
Principle of DTS.

The Distributed Temperature System (DTS) is based on the principle of Raman scattering, enabling temperature measurement by analyzing the interaction between laser pulses and the molecular structure of optical fibers. When a laser pulse is injected into the fiber, various scattering phenomena, including Rayleigh scattering, Brillouin scattering, and Raman scattering, are induced [36]. Among these, the Raman scattering is utilized for temperature detection. As the laser pulse propagates along the optical fiber, a portion of the scattered light is reflected back toward the source. The DTS system determines temperature by analyzing the intensity variations between the Stokes and anti-Stokes components of the backscattered Raman light. The operating principle of DTS is illustrated in Figure 1.

By measuring the time difference *t* between the incident light and scattered light, the distance *X* from the scattering point to the incident end can be determined using Equation (1). In the equation, *c* is the speed of light, expressed in meters per second (m/s).

(1) $$X=\frac{ct}{2}$$

The principle of Raman scattering involves the excitation of spontaneous Raman scattering within the optical fiber by a laser pulse [37]. This process generates two types of scattered light: Stokes, which has a longer wavelength than the incident light, and anti-Stokes, which has a shorter wavelength. The intensity of the anti-Stokes in the optical fiber is influenced by external temperature variations. The relationship between the ratio of anti-Stokes to Stokes intensity and temperature is given by Equation (2).

(2) $$\frac{I_{as}}{I_{s}}=\alpha e^{-\frac{hc∆v}{kT}}$$

In the equation, $I_{\mathrm{as}}$ is the anti-Stokes light intensity and $I_{s}$ is the Stokes light intensity. The parameter *α* is the temperature-dependent coefficient. h is Planck’s constant, expressed in joule-seconds J∙s, ∆v is the Raman frequency translation amount with units of $m^{-1}$, and k is Planck’s constant with units of J/K. The temperature can be calculated based on the ratio of the measured intensities of anti-Stokes and Stokes light, as follows in Equation (3).

(3) $$T=\frac{hc∆v}{k\left[\ln\alpha -\ln\left(\frac{I_{as}}{I_{s}}\right)\right]}$$

### 2.2. Temperature-Sensitive Concrete System

**Figure 2:**
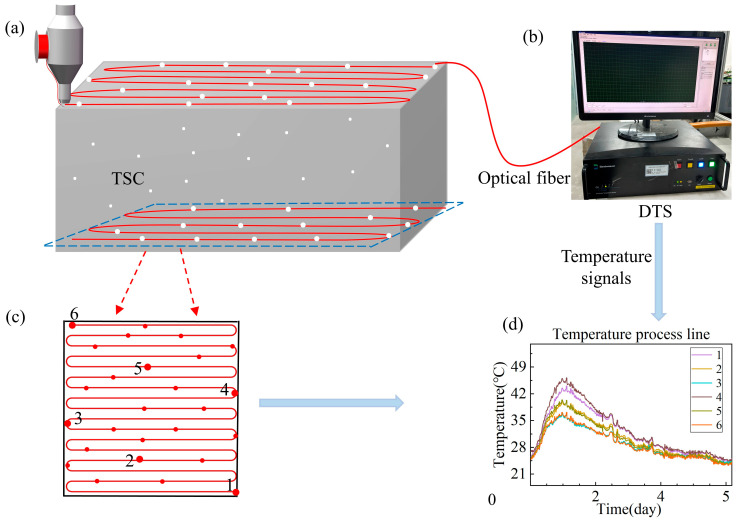
Temperature-sensitive concrete system. (**a**) Temperature-sensitive concrete. (**b**) Distributed temperature sensing. (**c**) Spatial distribution of 6 random monitoring points in concrete. (**d**) Temperature process line.

TSC is an innovative smart concrete system composed of distributed optical fibers embedded within the concrete matrix, as shown in Figure 2a. The distributed optical fiber is a continuous sensing line integrated into the concrete structure. The end of distributed optical fiber is connected to a DTS, which collects and records temperature data continuously in both space and time, as illustrated in Figure 2b. The TSC system consists of TSC and DTS, as illustrated in Figure 2. The temperature signals transmitted to the DTS are stored and processed to generate a temperature–time curve (Figure 2d). This system enables real-time monitoring of temperature within the concrete, ensuring accurate acquisition of temperature data (Figure 2c) and effectively tracking internal temperature field evolution (TFE) in real time.

## 3. Experimental Program

### 3.1. Mixture Design

The components used in TSC include 3DPC and distributed optical fiber. The raw materials for the concrete are divided into two categories: basic components and admixtures. The basic components comprise cementitious materials and fine aggregates. The cementing materials used are Ordinary Portland Cement, and the fine aggregate used is natural sand. The admixtures include concrete lubricant and polycarboxylate-based high-performance superplasticizer.

The Ordinary Portland Cement is P·O 42.5 grade cement (Yichang City, Hubei Province, China), a gray powder. Its primary mineral constituents are tricalcium silicate and dicalcium silicate. The fine aggregate is natural sand, predominantly composed of SiO₂. The sand is sieved through a 3 mm mesh to ensure all particles are smaller than 3 mm, with a well-graded particle size distribution. The chemical composition of cementing materials is shown in Table 1.

The concrete lubricant is a product from Dego Building Materials Co., Ltd. (Chengdu, Sichuan, China), consisting of sodium dodecyl sulfate and cellulose. The superplasticizer is produced by Mingzhu Waterproof and Corrosion Prevention Materials Co., Ltd. (Xinmi City, Henan Province, China). The physicochemical properties of the polycarboxylate-based high-performance superplasticizer are provided in Table 2. The mass ratio for TSC is shown in Table 3.

### 3.2. Optical Fiber Type

**Figure 3:**
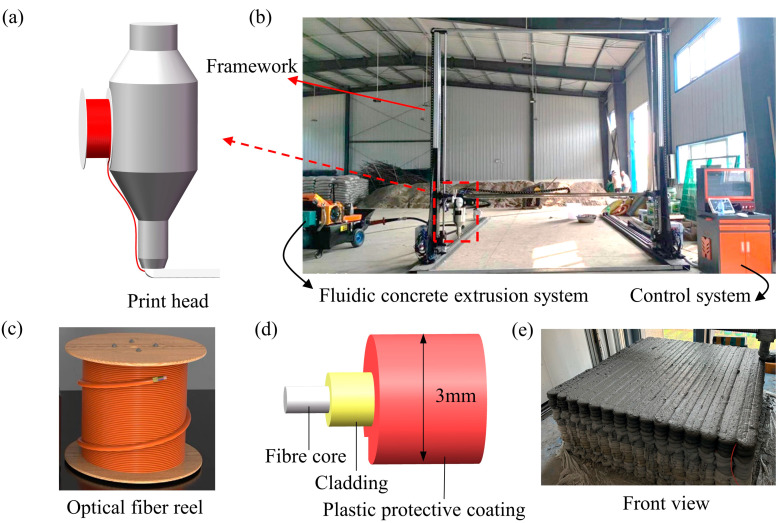
(**a**) Print head. (**b**) The integrated 3D printer for optical fibers and concrete. (**c**) Optical fiber reel. (**d**) The structure of the optical fiber. (**e**) Front view of 3D-printed temperature-sensitive concrete sample.

The distributed optical fiber, as a critical component of the TSCS, plays a vital role in data collection and transmission. Optical fibers are generally classified into single-mode and multi-mode types. Single-mode fibers support the transmission of light signals in only one mode, with light traveling in a straight path through the core without internal reflection. They offer a broad transmission bandwidth and high transmission speed, making them suitable for long-distance transmission. They are commonly employed in metropolitan area networks (MANs), wide area networks (WANs), long-distance communications, and data centers. Since the length of the optical fiber required for this experiment is less than 1500 m, a multi-mode fiber was selected. The optical fiber used in this study has a core diameter of 62.5 μm, a cladding diameter of 125 μm, a testing wavelength of 850 nm, a bending radius of 30 nm, and a tensile strength of 100 N. To prevent damage to the bare fiber during the experiment, a plastic protective layer with a diameter of 3 mm was applied to encase the fiber, ensuring its integrity when embedded within the concrete. The structure configuration of the optical fiber is shown in Figure 3d.

### 3.3. Concrete Mixing and 3D Printing

To prepare the TSC sample, a 3D printer capable of synchronously printing distributed optical fiber and concrete was designed and manufactured to achieve precise integration of fiber and concrete placement. Integrating distributed optical fiber and 3DPC printing technology has a large printing area, with a maximum printing size of 2.5 m × 2.5 m × 2.2 m and a printing nozzle diameter of 30 mm. The printing parameters are listed in Table 4. The 3D printer consists of four main components: the print head, the frame structure, the material handling system, and the control system. The print head enables synchronized deposition of both optical fibers and concrete. The material handling system is responsible for mixing, transporting, and extruding the concrete mixture. The control system co-ordinates the operation of the motors along each axis, facilitating multi-axis linkage and precise execution of the printing tasks. The overall structure of the integrated 3D printer is shown in Figure 3b.

## 4. Experimental Study on Temperature-Sensitive Concrete System

### 4.1. The Fabrication Method of Temperature-Sensitive Concrete Sample

To investigate temperature-sensitive 3DPC, a specialized sample was designed. The sample has dimensions of 1 m × 1 m × 0.5 m, with a layer height of 15 mm, a line width of 33 mm, and a total of 33 layers. The optical fiber reel (Figure 3c) was mounted at the feed port, and a 6 mm diameter feed tube was positioned adjacent to the nozzle of the concrete 3D print head. The optical fiber from the spool was guided through the feed tube, where it was held in place by the pressure of the extruded concrete at the tube outlet. The continuous movement of the extruder facilitated the synchronized deposition of both the optical fiber and the concrete material. This synchronicity in the laying of the optical fiber and concrete achieves the integrated printing technology, allowing each section of the printed concrete to be equipped with a sensing unit, thereby enabling precise monitoring of the temperature distribution.

### 4.2. Temperature Measurement Test of Temperature-Sensitive Concrete

The purpose of the temperature measurement test for TSC is to capture the dynamic and historical changes in the internal temperature field of concrete at different times and locations accurately. The DTS used in the test from Shanghai Bohui, DTS as shown in Figure 2b, has a positioning accuracy of 0.2 m and a temperature accuracy of ±1 °C. Before the experiment, the distributed optical fiber was connected to the temperature acquisition instrument, and necessary calibration of the sensor was performed. During the calibration process, key parameters such as sensor sensitivity and resolution were adjusted to ensure the accuracy of the measurements. Additionally, both the optical fiber and the temperature measurement instrument must undergo precision calibration to eliminate system errors and ensure the reliability of the temperature data. Upon completion of the calibration, the DTS was capable of displaying the real-time temperature distribution at any location within the concrete, providing accurate and reliable temperature monitoring data.

In this study, temperature data for each measurement point were recorded every 10 min, starting from the beginning of concrete printing, with continuous measurements taken over a period of 5 days. The selection of the 5-day monitoring period was based on the fact that the majority of the heat generated by the concrete’s hydration reaction is released within this timeframe. According to the experimental data, the hydration process is most active during the first 5 days, after which the hydration rate decreases substantially. Therefore, the temperature data collected over this period effectively capture the early-age behavior of the concrete and the influence of hydration heat. Although the 7-day curing period is commonly regarded as a significant reference point in concrete studies, the 5-day period was considered sufficient for evaluating the effects of hydration heat in this investigation.

**Figure 4:**
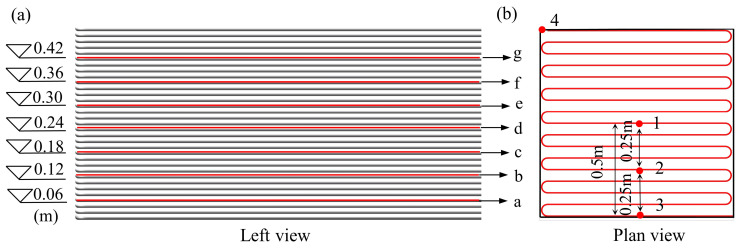
Sensing point layout. (**a**) Left view of the 3D-printed temperature-sensitive concrete sample. (**b**) Plan view of the measurement point layout.

A total of seven regions were defined within a height range of 0.5 m, labeled from Region a through Region g. The absolute heights for each region were 0.06 m, 0.12 m, 0.18 m, 0.24 m, 0.30 m, 0.36 m, and 0.42 m. A left-side view of the 3D-printed TSC specimen is shown in Figure 4a. In each region, four specific monitoring points were selected, resulting in a total of 28 measurement points. From the top view, these points were located at the center and distances of 0.25 m and 0.5 m from the center. The plan view of the measurement point layout is presented in Figure 4b. The selected monitoring points were designed to represent the temperature characteristics at various locations within the 3DPC. Measurement point 1, located at the center of the plane, recorded the highest temperature and effectively reflected the heat accumulation during the cement hydration process. The data variation from this point aids in analyzing the degree of hydration and thermal conductivity of the concrete. Measurement point 4, located at the farthest corner, is most significantly influenced by the external environmental temperature, and its readings represent the environmental temperature near the concrete surface. Measurement points 2 and 3, located along the horizontal axis between points 1 and 4, captured the effects of varying protective layer thicknesses on temperature transmission, thus providing data on the thermal transfer characteristics. Additionally, the selected monitoring points were evenly distributed across different heights and spatial locations, ensuring comprehensive representation of the dynamic temperature field both inside and near the surface of the concrete. This arrangement provides robust data support for analyzing the temperature behavior of 3DPC.

### 4.3. Test Results and Analysis

#### 4.3.1. The Influence of Ambient Temperature on Surface 3DPC Temperature

To validate the accuracy of the measured concrete temperature data, the temperature of the optical fiber positioned outside the concrete was measured by the DTS system at hourly intervals. These measurements were then compared with the environmental temperature recorded during the same period.

**Figure 5:**
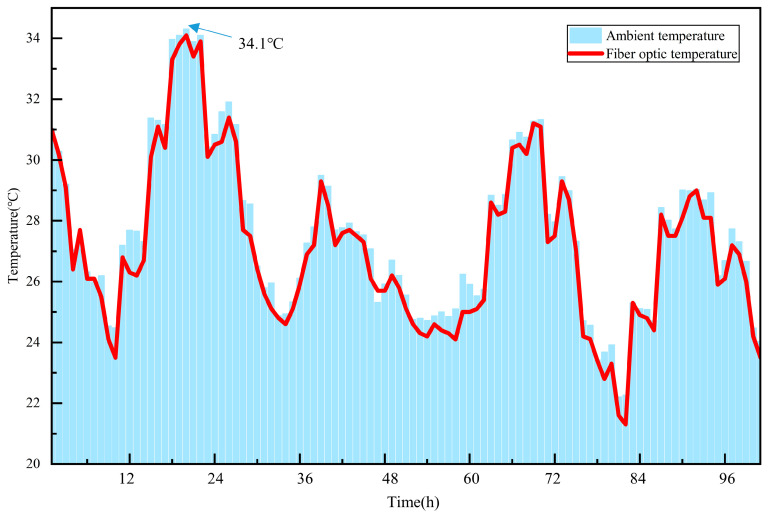
Comparison of environmental temperature and external concrete optical fiber temperature.

Continuous temperature monitoring of the TSC sample was conducted continuously for a 5-day age, beginning from the initiation of printing. During the initial phase of monitoring, the weather was predominantly clear, with the highest temperature reaching 34.1 °C. Subsequently, from the third to the fifth day, the temperature decreased significantly compared to the first two days, accompanied by notable fluctuations. The variations in environmental temperature and the temperature changes of the external fiber optic in the concrete are provided in Figure 5. The results indicate that the temperature curve recorded by the external optical fiber closely aligned with the environmental temperature, with deviations maintained within ±1 °C.

#### 4.3.2. Temperature Process Line Analysis for Different Locations

**Figure 6:**
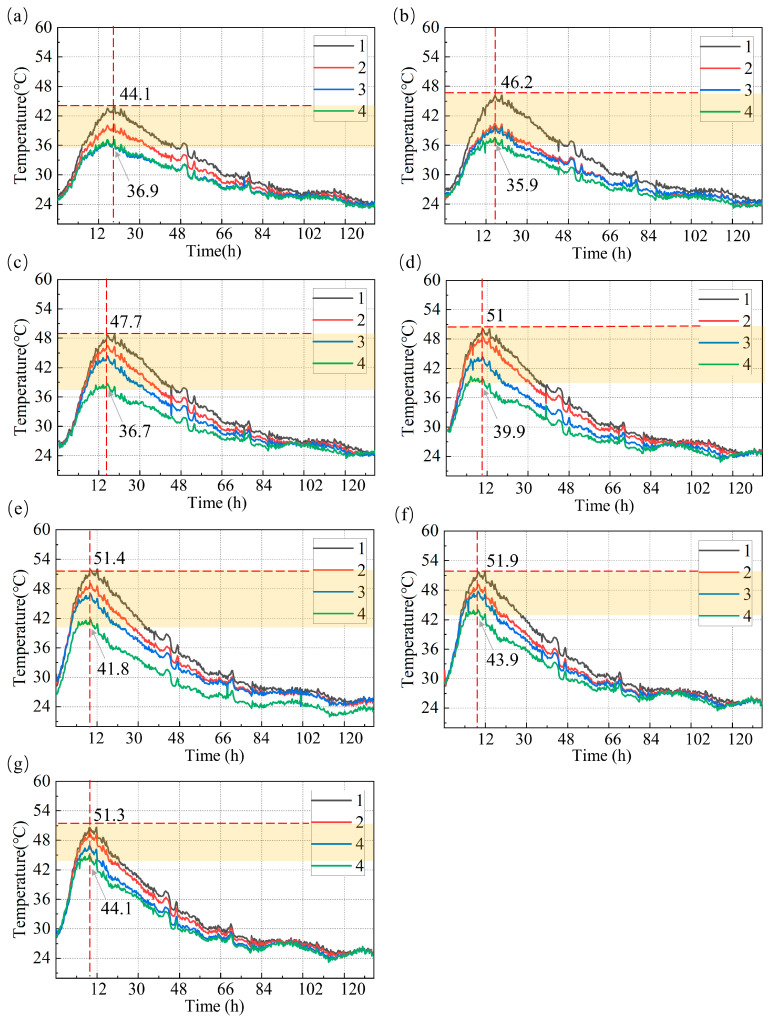
Temperature process lines of four special measurement points in seven different regions. (**a**) Region a. (**b**) Region b. (**c**) Region c. (**d**) Region d. (**e**) Region e. (**f**) Region f. (**g**) Region g.

Figure 6 presents the temperature variation curves for 28 monitoring points distributed across seven measurement regions. Based on the data shown, the temperature evolution characteristics of the concrete during the early curing processes can be observed. From Day 1 to Day 5, the temperature variations exhibit the typical behavior associated with the release of hydration heat.

In several subplots of Figure 6, the red dashed line appears around 12 h, marking a critical point in the hydration heat release process. At this stage, the rapid hydration reaction generates a significant amount of heat, resulting in a sharp increase in temperature. The subsequent temperature decline after the red dashed line indicates a slowdown in the release of hydration heat and the concrete temperature gradually stabilizes. This turning point highlights the relationship between hydration, heat release, and temperature field evolution. The red dashed line serves as a key time reference for further analysis of the concrete temperature field development.

In Figure 6, the yellow shaded areas represent the highest recorded temperatures at different monitoring points across various locations. The data indicate that the temperature at point 1 is consistently higher than that at point 2, while points 3 and 4 exhibit similar temperature profiles. Specifically, point 1 in Region f reaches the highest recorded temperature of 51.9 °C, located at the center of the concrete section. During hydration, the accumulation of heat in the concrete core results in elevated temperatures due to the rapid release of hydration heat and the relatively slow dissipation of heat to the surrounding environment, particularly in the central regions where heat transfer is less effective.

Furthermore, the temperature peaks recorded in Regions e to g are generally higher than those in Regions a to d. Specifically, the peak temperatures in Regions e, f, and g are 51.4 °C, 51.9 °C, and 51.3 °C, respectively (see Figure 6e–g), while the peaks in Regions a to d are 44.4 °C, 46.2 °C, 47.7 °C, and 51.0 °C, respectively (see Figure 6a–d). This phenomenon is primarily attributed to the influence of external environmental factors on the surface layers of the concrete in Regions e to g, where heat dissipation is less efficient, resulting in greater heat accumulation and higher temperature peaks. In contrast, Regions a to d exhibit relatively lower temperatures due to more effective heat dissipation conditions. After the critical time point marked by the red dashed line, temperatures in these regions decrease rapidly, demonstrating comparable cooling rates. This suggests that, in these regions, the concrete exhibits relatively high heat dissipation efficiency. As the rate of hydration heat release slows down, the temperature gradually decreases. These observations further indicate that the differences in external environmental conditions, hydration heat release, and heat dissipation conditions play significant roles in the evolution of the temperature field.

#### 4.3.3. Temperature Contour Map Analysis for Different Ages

**Figure 7:**
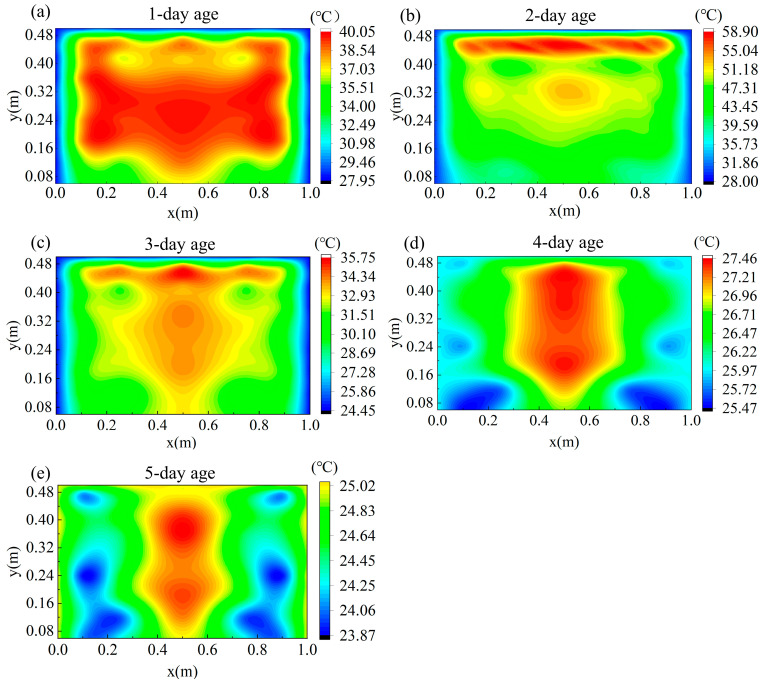
Temperature contour map of point 1 in different regions. (**a**) One-day age. (**b**) Two-day age. (**c**) Three-day age. (**d**) Four-day age. (**e**) Five-day age.

Figure 7 presents the distribution of hydration heat at the location of point 1 in the experimental data at a 5-day age. The figure illustrates the temporal evolution of the temperature field during the hydration process. It is a contour plot of the temperature field, where the horizontal axis represents the horizontal position and the vertical axis represents the vertical position. The colors in the figure represent different temperature values, with the range of temperatures indicated by the color scale on the right.

During the initial stage of hydration, the high-temperature region (red area) is primarily concentrated at the center of the concrete, where temperatures exceed 40 °C. This indicates that the heat generated by the hydration reaction is initially concentrated in the interior of the concrete. The surrounding temperature is lower, with a gradual outward decrease, displaying a radial temperature decay. As the hydration progresses, the temperature distribution becomes more uniform. The high-temperature area in the center gradually shrinks, with temperatures decreasing to around 35 °C, and the surrounding regions stabilize in temperature. By the 5-day age, the temperature field stabilizes, with temperatures ranging from approximately 24 °C to 25 °C, indicating that most of the hydration heat has dissipated and the internal temperature approaches ambient conditions.

The numerical simulation is based on an idealized heat transfer model. Although actual boundary conditions and the thermal properties of the materials were incorporated as accurately as possible, the thermal environment during the 3D printing process remains highly complex. Factors such as the printing path, fluctuations in ambient humidity, and material heterogeneity are difficult to fully account for in the model, resulting in localized discrepancies in the temperature distribution. Nevertheless, the overall trends predicted by the simulation align well with the experimental data, demonstrating that the model maintains good predictive capability at the macroscopic level and validating its applicability.

## 5. Numerical Simulation of Temperature-Sensitive Concrete System

### 5.1. Simulation of the Concrete Printing Process

**Figure 8:**
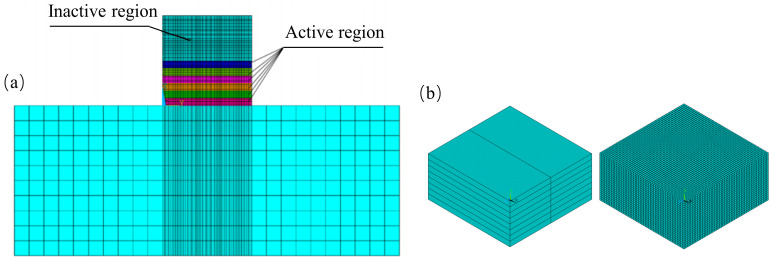
Simulation of the concrete printing process. (**a**) Schematic diagram of the 3D printing simulation process. (**b**) Model and mesh division.

This study simulates the layer-by-layer extrusion process of 3DPC, with consideration of the effect of printing speed on the activation of each unit in the model. In the simulation, each unit is activated on an hourly basis according to printing progress and assigned corresponding material properties, with the initial temperature set to the printing temperature of the concrete. The continuous extrusion of concrete from the printing nozzle is modeled as a step-by-step deposition process composed of hexahedral elements. Based on the specified printing speed, concrete units are progressively activated layer by layer along the nozzle path. A schematic diagram of the 3D printing simulation process is shown in Figure 8a.

In the simulation, the boundary conditions are defined as follows: a triaxial constraint is applied to the base rock surface, while normal constraints are applied to the four side surfaces. Additionally, adiabatic boundary conditions are assigned to the nodes on both the base and the four side surfaces. This condition ensures zero heat flux across the boundary, consistent with the assumption that the surrounding environment has negligible thermal influence during the short duration of the printing process. This setup effectively replicates the external mechanical and thermal constraints encountered by the concrete during the printing process, providing a more realistic representation of the heat conduction and hydration reactions during the actual printing process. The application of adiabatic boundary conditions is as follows.

At the initial time, the temperature field is a known function of the spatial co-ordinates (*x*, *y*, *z*), denoted as *T*_0_ (*x*, *y*, *z*), i.e., when τ = 0:

(4) $$T\left(x,y,z,0\right)=T_{0}\left(x,y,z\right)$$

The heat flux on the concrete surface is a known function of time, i.e.:

(5) $$-\lambda \frac{\partial T}{\partial n}=f\left(\tau \right)$$

*n* represents the outward normal direction of the surface. In the case of an adiabatic boundary, the following condition must be satisfied:

(6) $$\frac{\partial T}{\partial n}=0$$

In the simulation of this 3D-printed TSC, the model consists of uniformly sized poured cubic blocks, with actual boundary conditions and material parameters during the pouring process. After establishing the concrete model, finite element meshing was applied. The basic mesh size was set to 0.06 m, while the mesh in the concrete part was refined to 0.02 m. The model and mesh division are shown in Figure 8b.

### 5.2. Material Parameters

The density, thermal conductivity, specific heat capacity, and other simulation parameters used in this study were adopted from the values employed by Ma et al. [38] in their analysis of temperature stresses in 3DPC. The specific thermophysical properties of the concrete are shown in Table 5. During the numerical simulation, the positions of the simulated nodes corresponded to the measurement points obtained from the experimental data. Ultimately, the temperature process lines and temperature contour maps were generated based on the simulation results.

The elastic modulus of the printed concrete is expressed in the form of a composite exponential function:

(7) $$E\left(\tau \right)=25.3\left(1-e^{-0.009\tau^{0.789}}\right)$$

where *E*(*τ*) is the elastic modulus of the concrete (GPa), and *τ* is the age (hours). The adiabatic temperature rise fitting formula also adopts a composite exponential form:

(8) $$Q\left(\tau \right)=40\left(1-e^{-0.07\tau^{0.61}}\right)$$

where *Q*(*τ*) is the adiabatic temperature rise of the concrete (°C), and τ is the age (hours).

### 5.3. Results and Analysis

#### 5.3.1. Results Comparison of Experimental and Simulation Temperatures

**Figure 9:**
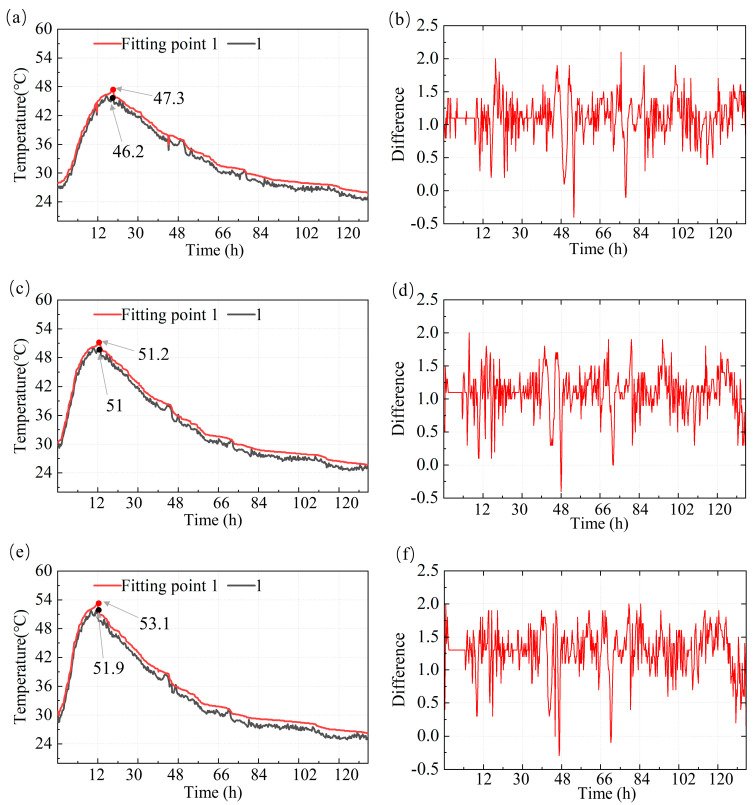
Comparison of temperature process lines and numerical simulation error for measurement point 1 in different regions. (**a**) Temperature process line for region b. (**b**) Error analysis for region b. (**c**) Temperature process line for region d. (**d**) Error analysis for region d. (**e**) Temperature process line for region f. (**f**) Error analysis for region f.

Figure 9a,c,e show the experimental data (black curve) and numerical simulation data (red curve) of temperature process lines over time in different concrete regions (b, d, and f). By examining these curves, it is evident that the experimental data and numerical simulation data exhibit a high degree of consistency in their overall trends.

Figure 9b,d,f present the differences between the experimental measurements and numerical simulation results. These plots illustrate the fluctuations and deviations over time between the two datasets. During the initial temperature rise phase, from Day 1 to Day 5, the temperature differences remain relatively small, generally within the range of 0 °C to 1.0 °C. This indicates that the numerical model accurately captures the heat release behavior associated with the hydration process.

As time progresses, particularly after 66 h, when the concrete temperature begins to stabilize, the fluctuations in the differences become more pronounced. The maximum deviation occurs around 66 h, reaching up to 2 °C. These discrepancies may result from assumptions made in the simulation process, including boundary conditions related to heat convection and conduction, as well as potential errors in experimental data acquisition.

The high-frequency fluctuations observed in the difference plots may reflect the dynamic nature of hydration heat release during the later stages, particularly when elevated temperatures and variations in heat conduction are present. At this stage, the numerical simulation may not fully account for the rapid changes in hydration heat generation or may exhibit slight delays in the predicted temperature response.

Overall, the comparison between the numerical simulation and experimental data demonstrates that the simulation model provides accurate predictions of the concrete temperature field, particularly during the stabilization phase. Although some deviations are observed during the rapid heat release phase of hydration, these differences remain within an acceptable range. The deviations in the numerical simulations are generally controlled within 2 °C, which is considered acceptable for predicting the overall temperature distribution and assessing the risk of early-age cracking in concrete structures.

#### 5.3.2. Comparison of Temperature Fields Between Numerical Simulation and Experimental Data

**Figure 10:**
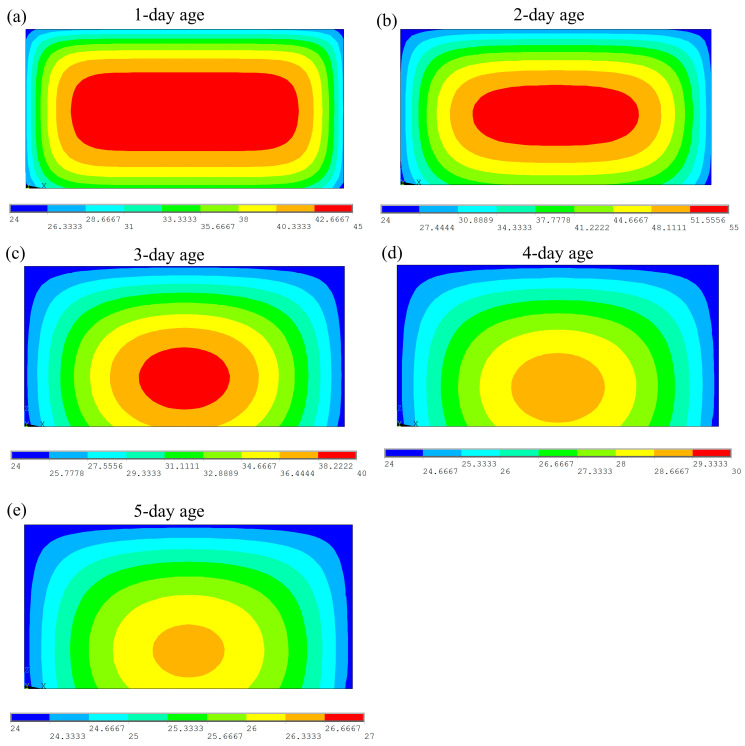
Evolution of the temperature field at different ages in numerical simulation. (**a**) One-day age. (**b**) Two-day age. (**c**) Three-day age. (**d**) Four-day age. (**e**) Five-day age.

Figure 10 shows the temperature distribution at the location of point 1 at a 5-day age, obtained through numerical simulation. The temperature field is illustrated using a contour plot. The numerical simulation results indicate that the effect of the hydration heat release process on the temperature field is consistent with the trend observed in the experimental data shown in Figure 7.

During the early stages of the simulation, the high-temperature region is primarily concentrated at the center of the concrete, where temperatures exceed 40 °C. This indicates that hydration heat release is initially concentrated in the core region. As the simulation progresses, the temperature gradually extends to lower regions and the central high-temperature zone shrinks, with the temperature dropping to approximately 35 °C. After 5 days of hydration, the temperature field stabilizes, with the overall temperature decreasing to around 24 °C, which aligns with the stable temperature values observed in the experimental data.

The numerical simulation is based on an idealized heat conduction model. Although the actual boundary conditions and the thermophysical properties of the materials were incorporated as accurately as possible, the thermal environment during the 3D printing process is highly complex. Factors such as the printing path, fluctuations in ambient humidity, and material heterogeneity are difficult to fully capture within the model, resulting in localized discrepancies in the temperature distribution. Nevertheless, the overall trend predicted by the simulation is consistent with the experimental measurements, demonstrating that the model maintains good predictive capability at the macroscopic level and validating its reliability.

## 6. Conclusions

A novel method for TSC is proposed, in which the integration of distributed optical fiber and 3DPC technology enables each point within the concrete to incorporate a sensing unit capable of collecting, storing, and transmitting temperature signals. To validate the effectiveness of this approach, both experimental demonstrations and numerical simulations were conducted for the TFE in a 3D-printed optical fiber concrete cubic sample. The experimental program demonstrated the collection, storage, and transmission of TFE data in real time. Meanwhile, the numerical simulation computed the TFE based on the thermal conduction equation and the actual boundary conditions during the printing process of the cubic sample. The temperature field at each time step and the temperature history at each monitoring point were obtained. The experimental results were compared with the numerical simulation. The following conclusions can be drawn:

(1) The proposed innovation and theory of TSC prove viable to monitor TFE by integrating distributed optical fiber and 3DPC technology. Three-dimensional printing technology is introduced to integrate organic optical fiber and concrete everywhere. Distributed optical fiber is used for temperature sensing and signal transmission. This enables TSC to monitor and record its internal temperature distribution in real time, facilitating structural health monitoring and environmental condition analysis.

(2) The temperature curve obtained from the optical fiber monitoring system closely matches the ambient temperature, with fluctuations maintained within ±1 °C. This indicates that the integrated 3D printing and optical fiber sensing system demonstrates high accuracy and stability in monitoring the temperature distribution of the concrete.

(3) The experimental tests and numerical simulations of the TFE in the 3D-printed optical fiber concrete cubic sample show a high degree of consistency, with deviations limited to within 5%. This confirms the feasibility and effectiveness of the proposed method for practical engineering applications.

In summary, this study presents an innovative approach that integrates distributed optical fiber sensing technology with 3DPC to develop an intelligent temperature-sensitive concrete monitoring system. The proposed method effectively addresses the limitations of existing technologies in real-time temperature monitoring of large-scale concrete structures under complex environmental conditions, providing a novel solution for the early detection and monitoring of temperature-induced cracking.

Although 3D printing technology offers significant advantages for the integrated embedding of distributed optical fibers within concrete, enhancing system integration, improving physical protection for the sensors, and reducing the risk of damage caused by improper manual installation, several challenges remain. First, the design of the printing path plays a critical role in determining the overall performance of the monitoring system. Inappropriate path planning may lead to anisotropy in the internal structure of the printed concrete, adversely affecting the uniformity of heat transfer and compromising the stability of signals within the distributed optical fiber sensing system. Second, under long-term service conditions, the system remains susceptible to extreme environmental factors, such as sustained high humidity and other harsh conditions. These factors may cause signal attenuation or even disruption of the optical fibers, thereby reducing the accuracy of temperature monitoring and the long-term reliability of the system. At present, the system has not yet fully overcome the challenges posed by such extreme environmental conditions.

Based on the above analysis, future research should focus on optimizing the 3D printing process parameters and path planning to minimize the potential negative impacts of the printing process on the performance of the sensing system. In parallel, further refinement of the concrete mix design is essential to enhance the adaptability and durability of the integrated system. Moreover, a comprehensive cost–benefit analysis is required to systematically evaluate the engineering feasibility and economic viability of the proposed temperature monitoring system in large-scale civil infrastructure applications, such as bridges and dams. These efforts will provide a solid theoretical foundation and empirical data to support the practical implementation and broader adoption of this technology. Overall, this study presents a novel technical pathway for the development of intelligent concrete and offers valuable support for advancing smart construction in building and infrastructure engineering.

## Figures and Tables

**Table 1:** Chemical composition of cementing materials (wt.%).

Components	AL_2_O_3_	SiO_2_	Fe_2_O_3_	CaO	SO_3_	K_2_O	MgO	TiO_2_
Cement	5.26	22.65	3.33	59.12	2.71	0.81	2.21	0.52
Sand	<1	85	<1	<1	-	-	<1	-

**Table 2:** Physicochemical properties of polycarboxylate-based high-performance superplasticizer.

Density (g/cm^3^)	PH Value (23 °C, 40% Concentration)	Total Chloride Ion Content	Alkali Content
0.40~0.60	9.0~12.0	≤0.1%	≤3.0%

**Table 3:** Mass ratio for temperature-sensitive concrete (kg/m^3^).

Cement	Sand	Water	Cement Mortar Lubricant	Accelerator
634.29	1268.38	253.68	2.54	41.22

**Table 4:** Printing parameter settings.

Layer Height (mm)	Print Width (mm)	Fill Density (%)	Fill Pattern	Print Speed (mm/s)	Extrusion Flow Rate (m^3^/s)
15	33	100	Concentric circles	50	2.3

**Table 5:** Thermodynamic parameters of bedrock and concrete.

Parameters	3D-Printed TSC
Bulk density, *γ*/kg·m^−3^	2350
Specific heat, *C*/kJ·(kg·°C)^−1^	1.007
Thermal conductivity, *λ*/kJ·(m·h·°C)^−1^	9.56
Thermal diffusivity, *a*/m^2^·h^−1^	0.0040
Ultimate adiabatic temperature rise, *θ*_0_/°C	49
Poisson’s ratio	0.17
Coefficient of linear expansion, *α*/10^−6^·°C^−1^	7
Ultimate elastic modulus, *E*_0_/GPa	30

## Data Availability

The original contributions presented in this study are included in the article. Further inquiries can be directed to the corresponding author.

## Abbreviations

The following abbreviations are used in this manuscript:

3DPC	Three-dimensional printing concrete
TSC	Temperature-sensitive concrete
TSCS	Temperature-sensitive concrete system
DTS	Distributed Temperature Sensing
TFE	Temperature field evolution
